# Variation in loss of immunity shapes influenza epidemics and the impact of vaccination

**DOI:** 10.1186/s12879-017-2716-y

**Published:** 2017-09-19

**Authors:** Rutger G. Woolthuis, Jacco Wallinga, Michiel van Boven

**Affiliations:** 1 Theoretical Biology, Utrecht University, Padualaan 8, Utrecht, 3584 CH The Netherlands; 2 National Institute for Public Health and the Environment, Antonie van Leeuwenhoeklaan 9, Bilthoven, 3721 MA The Netherlands

## Abstract

**Background:**

Protective antibody immunity against the influenza A virus wanes in 2–7 years due to antigenic drift of the virus’ surface proteins. The duration of immune protection is highly variable because antigenic evolution of the virus is irregular. Currently, the variable nature of the duration of immunity has had little attention in analyses of the impact of vaccination, including cost-effectiveness studies.

**Methods:**

We developed a range of mathematical transmission models to investigate the effect of variable duration of immunity on the size of seasonal epidemics. The models range from simple conceptual to more realistic, by distinguishing between infection- versus vaccination-induced immunity, by inclusion of primary vaccine failure, by assuming a leaky vaccine, and by the inclusion of age-dependent contact patterns.

**Results:**

We show that annual variation in the duration of immunity causes large variation in the size of epidemics, and affects the effectiveness of vaccination. Accumulation of susceptible individuals in one or more mild seasons results in a disproportionately large outbreak in a subsequent season. Importantly, variation in the duration of immunity increases the average infection attack rate when the vaccination coverage is around the outbreak threshold. Specifically, in a tailored age-stratified model with a realistic reproduction number (*R*_0_ = 1.4) and vaccination coverage of 25%, we find that the attack rate in unvaccinated children (<10 years old) is negligible if the duration of immunity is constant, while on average 2.8% (2.5–97.5% percentiles: 1.8–4.1%) of the children are infected if the duration of immunity is variable. These findings stem from the buildup of susceptibility over multiple seasons by waning of immunity, and the nonlinear relation between susceptibility and infection attack rates.

**Conclusions:**

The models illustrate that variation in the duration of immunity impacts the long-term effectiveness of vaccination, and that vaccine effectiveness cannot be judged for each year in isolation. Our findings have implications for vaccination strategies that aim to maximize the vaccination coverage while extending the age range of persons eligible for vaccination.

**Electronic supplementary material:**

The online version of this article (doi:10.1186/s12879-017-2716-y) contains supplementary material, which is available to authorized users.

## Background

The influenza virus is responsible for a substantial health burden with annual infection attack rates of 5–10% in adults and 20–30% in children [1]. Annual vaccination can protect against influenza infection and therefore vaccination is recommended for elderly and other individuals at high-risk of developing serious disease [2–4]. Despite efforts to improve vaccines against influenza, vaccination still provides only partial protection, and antibodies generated after infection or vaccination protect a person only for a limited number of years. This is because the evolution of influenza’s surface proteins prevents neutralization of evolved viruses by preexisting antibodies. This so-called antigenic drift is irregular [5], with relative conserved periods that last 2–7 years [6–8], and is only modestly predictable [9, 10]. The variable duration of immunity is a cause of substantial year-to-year differences of estimates of vaccine efficacy and vaccine effectiveness [11–16], illustrating the need for robust models to assess vaccination policies.

Most epidemiological and cost-effectiveness analyses of influenza vaccination assume that the rate at which immunity is lost does not vary between years [17–33]. In this were the case, each influenza season could be analyzed in isolation. In the more realistic scenario of a variable rate at which immunity is lost due to antigenic drift, the fraction of the population that is susceptible (henceforth susceptibility of the population) will vary between years. In this case, it is necessary to track the number of infections that happened in previous seasons, and that still give protection against the currently circulating virus. The importance of linking the infection history of successive seasons is recognized [34, 35], but difficult to implement in prospective studies [36], since the level of preexisting immunity in the population as well as the antigenic evolution of the virus are hard to predict.

Here we study the impact of variation in the duration of immunity on the infection attack rates and the epidemic peaks in a disease transmission framework. To focus on the key elements of acquired immunity, vaccination, and influenza evolution, we formulate an idealized model in which the susceptibility in each season depends on the cumulative effect of all infections and vaccinations in the past. We show that variation in the duration of immunity results in alternating mild and severe seasons, and in an increase in the average infection attack rate and peak prevalence. Especially around the critical vaccination coverage, we find that variation in the duration of immunity increases the number of infections. We also show that these findings are not restricted to highly simplified models, but are also observed in more realistic influenza transmission models.

## Methods

### Overview

For each season we calculate population susceptibility using the accumulation of influenza infections and influenza vaccinations over previous years [37]. Susceptibility increases due to waning of immunity and demographic turnover, and decreases by vaccination and infection. Throughout, we focus on populations in temperate zones where epidemics generally last for 4–12 weeks, and where there is hardly influenza circulation in the remainder of the year [38]. In our models, vaccination takes place just before the epidemic, while loss of immunity occurs after the epidemic when there is no virus circulation. Demographic turnover is not separately modeled, as births increase the number of susceptible persons in a similar fashion as loss of immunity does if population size is constant. We map susceptibility at the start of the season to the attack rate using the reproduction number, which is defined as the average number of infections caused by one infected individual in a fully susceptible population (as described in more detail below). Throughout, we compare scenarios with constant and variable duration of immunity. In the variable immunity model, the fractions of the immune individuals that stays immune in the next season are drawn from a Beta distribution that mimics the punctuated antigenic drift of the H3N2 subtype observed using hemagglutination inhibition tests [6, 7].

Below we formulate a basic model to show that a variable duration of immunity increases the epidemic peaks and infection attack rates. We then adapt the basic model so that vaccination provides protection for one season. Finally, we consider three extensions with (i) a ‘leaky’ vaccine, (ii) an age-structured population model with age-specific contact patterns, and (iii) realistic latent and infectious period distributions in an age-structured population with leaky vaccination [35]. The extensions show that the phenomena observed in the basic model are robust, and do not depend on specific model assumptions.

### Model structure

In our basic model, individuals are either fully susceptible to infection or fully immune, either due to natural infection or vaccination. The fraction of susceptible individuals (susceptibility) is determined by the cumulated immunity during outbreaks and vaccination in past seasons. Three processes link the susceptibility *s*_*t*+1_ in season *t*+1 to the susceptibility *s*_*t*_ in the previous season, i) vaccination, ii) the yearly influenza epidemic, and iii) the loss of immunity during the inter-epidemic period by virus evolution and demographic turnover. As these processes largely take place sequentially and on different timescales, we model these processes sequentially. In the model, vaccination is applied before the epidemic, and loss of immunity takes place after the epidemic in the inter-epidemic period. Schematically, the model looks as follows:

$$ s_{t} \xrightarrow{\text{vaccination}} s{\prime}_{t} \xrightarrow{\text{epidemic}} s{\prime}{\prime}_{t} \xrightarrow{\text{waning immunity}} s_{t+1}~.   $$

In the first step, a fraction of the population is vaccinated, such that the fraction remaining susceptible after vaccination is given by,

1 $$ s{\prime}_{t}=\left(1-v_{c}v_{e}\right)s_{t},   $$

where *v*_*c*_ and *v*_*e*_ are the vaccination coverage (the proportion of individuals who are vaccinated) and vaccine efficacy (the proportion of vaccinated individuals that is protected against infection), respectively. Thus, a fraction *v*_*c*_ *v*_*e*_ is protected against infection. Throughout, we vary *v*_*c*_ and take *v*_*e*_=0.5. To keep the model simple and make our arguments as transparent as possible, we assume that each year vaccination is given to a random set of individuals.

Next, part of the population is infected during the epidemic, and will be immune afterward:

$$s{\prime}{\prime}_{t}=s{\prime}_{t}-z_{t}, $$

where *z*_*t*_:=*z*_*t*_(*s*) is the attack rate (the fraction infected in season *t*). In a fully susceptible population (*s*=1) the attack rate is given by *z*_*t*_=1− exp(−*z*_*t*_ *R*_0_), with *R*_0_ representing the basic reproduction number, defined as the expected number of infections caused by one infected individual in a completely susceptible population. Estimates of the reproduction number for influenza (*R*_0_) range between 1-2 [38, 39]. Here, we take *R*_0_=1.4 as default value.

Due to immunity acquired in previous years, only a fraction *s* ^′^ is susceptible, such that

2 $$ z_{t}=s{\prime}(1-\exp(- z_{t} R_{0})).  $$

In the last step, immunity is lost, so that

$$s_{t+1}=1-\gamma_{t}(1-s{\prime}{\prime}_{t}), $$

where *γ*_*t*_ is the fraction of the population that retains its immunity. Loss of immunity is caused by the reduction of cross-protection between the current strain and strains circulating previous seasons. Throughout, we explore the impact of annual variation in the rate at which immunity is lost due to antigenic drift of the virus (i.e. the effect of variability in *γ*_*t*_). In the deterministic scenario we use $\gamma =\frac {5}{7}$, and in the stochastic scenario we draw *γ* from a realistic Beta(5,2) distribution that has a mean of 5/7. In this manner, the deterministic scenario arises naturally as a special case of the stochastic scenario when the parameters of the beta distribution tend to infinity. In both scenarios, immunity lasts 3.5 years on average in both scenarios.

Combining the above, we obtain the following set of equations that map the susceptibility *s*_*t*_ in year *t* to the susceptibility *s*_*t*+1_ in year *t*+1:

3 $$\begin{array}{*{20}l} z_{t} &= \left(1-v_{c}v_{e}\right) s_{t} \left(1-\exp\left(- z_{t} R_{0}\right)\right), \end{array} $$

$$\begin{array}{*{20}l} s_{t+1} &= 1-\gamma_{t} \left(1-\left(1-v_{c}v_{e}\right) s_{t} + z_{t}\right). \end{array} $$

These equations can be solved numerically.

The map in Eq. 3 is used to calculate the infection attack rate, but does not yield the infection prevalence over time (the epidemic curve). For this, we use the dynamical system corresponding to the above model. The ordinary differential equations (ODEs) for the fractions of the population in the susceptible (*S*), infected and infectious (*I*), and recovered (*R*) compartments during the epidemic are given by

$$\begin{array}{*{20}l} \frac{dS}{dt} & = - \beta S I, \\ \frac{dI}{dt} & = \beta S I - \nu I, \\ \frac{dR}{dt} & = \nu I. \end{array} $$

**Fig. 1:**
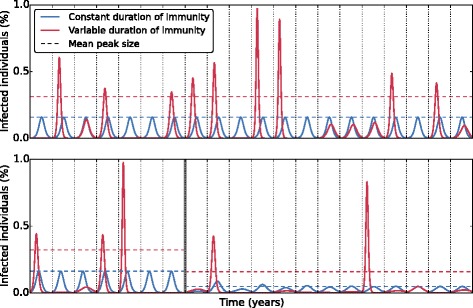
Variation in the duration of immunity increases the height of epidemic peaks. In the absence of vaccination, a pattern of regular epidemics is observed if the duration of immunity is constant (blue line), while an irregular pattern with alternating small and large epidemics is observed if the duration of immunity is variable (red line). After the introduction of vaccination (vertical gray line), the pattern of regular epidemics persists in the constant immune duration scenario, albeit at a lower level (blue line). If the duration of immunity is variable, the irregular pre-vaccination pattern of alternating small and large epidemics is exacerbated (red line). The mean of the prevalence peaks is substantially higher in the variable immunity scenario than in the constant immune duration scenario (dotted lines). The means of the prevalence peaks are calculated over 1,000 seasons using a burn-in period of 50 years. See Methods for details

Throughout, we fix *ν* and set *β*=*ν* *R*_0_, such that the infection attack rate is independent of *ν*. In Fig. 1 we take *ν*=2 (day^−1^) and *S*(0)=*s*′, *I*(0)=0.000001, and *R*(0)=1−*S*(0)−*I*(0), where *s*′ is the susceptibility after vaccination as given in Eq. 1. The differential equations are solved with a standard ODE solver using Python.

### Short-term protection of vaccination

Natural immunity (i.e. immunity acquired after infection) is likely to last longer than immunity provided by vaccination. In the basic model presented above (Eq. 3), we assume that vaccination gives the same duration of protection as natural infection. Here we present the other extreme that vaccination protects individuals for one season only. In this case the the model equations are given by

$$z_{t} = \left(1-v_{c}v_{e}\right) s_{t} \left(1-\exp\left(- z_{t} R_{0}\right)\right), $$

$$s_{t+1} = 1-\gamma_{t} \left(1 - s_{t} + z_{t}\right), $$

where only the attack rate depends on vaccination.

### Leaky vaccine model

It is known that influenza vaccines provide partial protection against infection and disease [12–14]. We therefore extend the basic model to include the possibility that the vaccine gives partial protection while infection still gives full protection. In this model, protection after infection and vaccination are assumed to wane at identical rates, while a vaccinated individual who is subsequently infected gains full protection.

In an all-or-nothing vaccine model, *v*_*e*_ is the proportion of vaccinated individuals that is protected, while with a leaky vaccine, we define *v*_*r*_ to be the probability that vaccinated individuals are protected in a single exposure that would have led to transmission to an unvaccinated individual. Unless stated otherwise, we take *v*_*e*_=1.0, and *v*_*r*_=0.5.

In the leaky vaccine model we need to track the fractions of the population that are susceptible (*s*) and vaccinated (*p*, partially immune). Following the same steps as before, we obtain

$$\begin{array}{*{20}l} {}s_{t} \!\rightarrow\! s{\prime}_{t}&\,=\,(\!1\,-\,v_{c} v_{e})s_{t} \!\rightarrow \!s{\prime}{\prime}_{t}\!=s{\prime}_{t} \,-\, z^{s}_{t} \!\rightarrow \!s_{t+1}\,=\,1-\gamma_{t}(1-s{\prime}{\prime}_{t}),\\ {}p_{t} \!\rightarrow \!p{\prime}_{t}&\,=\,p_{t}\,+\,v_{c} v_{e} s_{t} \rightarrow\! \!p{\prime}{\prime}_{t}\,=\,p{\prime}_{t} \,-\, z^{p}_{t}\! \rightarrow\!\! p_{t+1}=\gamma_{t} p{\prime}{\prime}_{t}~, \end{array} $$

where $z^{s}_{t}$ and $z^{p}_{t}$ are the attack rates of susceptible and vaccinated individuals, and $z_{t}=z^{s}_{t}+z^{p}_{t}$ is the overall attack rate. As in the basic model, each year a fraction 1−*γ*_*t*_ of the vaccinated and partially immune individuals lose immunity.

Combining the above, we obtain the following map linking *s*_*t*_ and *p*_*t*_ to *s*_*t*+1_ and *p*_*t*+1_:

4 $$\begin{array}{*{20}l} s_{t+1} & = 1-\gamma_{t} \left(1 - \left(1-v_{c} v_{e}\right)s_{t} + z^{s}_{t} \right)\\ p_{t+1}&=\gamma_{t} \left(p_{t} + v_{c} v_{e} s_{t}-z_{t}^{p} \right) \\ z^{s}_{t} & = s'_{t} \left(1 - \exp\left(-R_{0} \left(z^{s}_{t} + z^{p}_{t}\right)\right)\right)\\ z^{p}_{t} & = p'_{t} \left(1 - \exp\left(-R_{0} \left(1-v_{r}\right) \left(z^{s}_{t} + z^{p}_{t}\right) \right)\right)~.  \end{array} $$

As before, the above equations are readily solved numerically.

In a similar vein as before, we obtain a system of ODEs for the fractions of the population in the susceptible (*S*), partially protected (*P*), infected and infectious (*I*), and protected (*R*) compartments:

$$\begin{array}{*{20}l} \frac{dS}{dt} & = - \beta S I \\ \frac{dP}{dt} & = - \beta (1-v_{r}) P I \\ \frac{dI}{dt} & = \beta (S + (1-v_{r}) P) I - \nu I \\ \frac{dR}{dt} & = \nu I~, \end{array} $$

with initial conditions *S*(0)=*s* ^′^, *P*(0)=*p* ^′^, *I*(0)=0.000001, and *R*(0)=1−*S*(0)−*P*(0)−*I*(0).

### Age-stratified model

We extend the main model (Eq. 3) in another direction to include age structure. We consider three age classes: children (≤ 10 years), adults (> 10 and ≤ 60 years), and elderly (> 60 years). These broad classes are chosen to reflect the fact that young children are the drivers of transmission, while serious disease occurs mostly in elderly. Moreover, in many countries healthy elderly are eligible for vaccination (usually > 60 or > 65 years). The attack rates, with *i*∈ {children, adults, elderly}, are given by

5 $$ z_{t,i} = s{\prime}_{t,i} \left(1 - \exp \left(- R_{0} \sum_{j} g_{ij} z_{t,j} \right) \right).  $$

In the above, the next generation matrix ***G*** with elements *g*_*ij*_ is given by

$$g_{ij}=q f_{j} c_{ij}, $$

where *c*_*ij*_=*c*_*ji*_ is the (symmetric) contact rate between individuals in age groups *i* and *j*, and *f*_*j*_ is the proportion of the population in age group *j* [40]. The proportionality parameter *q* is used to scale ***G*** such that the dominant eigenvalue equals 1 and *R*_0_ in Eq. 5 corresponds to the basic reproduction number.

For simplicity, we assume a uniform population demography and lifespan of 80 years, such that ***f***=(1/8,5/8,2/8). Contacts between children are intense, while children and elderly have fewer contacts. In our analyses we take the following contact matrix

$$\boldsymbol{C}=\left(\begin{array}{ccc} 9 & 3 & 1\\ 3 & 3 & 2 \\ 1 & 2 & 2 \end{array}\right)~, $$

which is roughly in agreement with the contact structure as observed in representative contact survey studies in the Netherlands [40], and elsewhere [41].

The susceptibilities *s*_*t*+1,*i*_ in the next season are given by

$$\begin{array}{*{20}l} s_{t+1,i} & = 1-\gamma_{t} \left(1 - \left(1-v_{c}v_{e}\right) s_{t,i} + z_{t,i} \right), \end{array} $$

with *z*_*t,i*_ as in Eq. 5.

To calculate the peak prevalence in the age-stratified model (Eq. 5), we consider the age-stratified SIR-model with next-generation matrix ***G***, i.e.

$$\begin{array}{*{20}l} \frac{dS_{i}}{dt} & = - \beta S_{i} \sum_{j} g_{ij} I_{j} \\ \frac{dI_{i}}{dt} & = \beta S_{i} \sum_{j} g_{ij} I_{j} - \nu I_{i} \\ \frac{dR_{i}}{dt} & = \nu I_{i}, \end{array} $$

where *i,j*∈ {children, adults, elderly}.

### Tailored influenza transmission model

As a final extension, we consider a tailored transmission model that incorporates both age-dependent contact patterns and a partially immune compartment. In addition, we include compartments for latently infected infected individuals (i.e. those who are infected but not yet infectious), and assume that both the latent and infectious periods are gamma (Erlang) distributed. Such dynamical systems form the backbone of many influenza transmission models [35, 42]. Denoting the forces of infection by *λ*_*i*_ (*λ*_*i*_∈{children, adults, elderly}), the model dynamics during the epidemic are specified by the following system of ODEs:

$$\begin{array}{*{20}l} \frac{dS_{i}}{dt} & = - \lambda_{i} S_{i}, \\ \frac{dP_i}{dt} &= -(1-v_r) \lambda_{i} P_{i},\\ \\ \frac{dE^{(1)}_{i}}{dt} & = \lambda_{i} (S_{i} + (1-v_{r})P_{i}) - \nu E^{(1)}_{i},\\ \frac{dE^{(2)}_{i}}{dt} & = \nu E^{(1)}_{i} - \nu E^{(2)}_{i},\\ \\ \frac{dI^{(1)}_{i}}{dt} & = \nu E^{(2)}_{i} - \mu I^{(1)}_{i},\\ \frac{dI^{(2)}_{i}}{dt} & = \mu I^{(1)}_{i} - \mu I^{(2)}_{i},\\ \\ \frac{dR_{i}}{dt} & = \mu I^{(2)}_{i}, \end{array} $$

with forces of infection *λ*_*i*_ given by,

$$\lambda_{i} = \beta \sum_{j} g_{ij} \left(I^{(1)}_{j} + I^{(2)}_{j}\right), $$

and *i*,*j*∈ {children, adults, elderly}. Following [35], we take *ν*=2.5 (day ^−1^) and *μ*=1.1 (day ^−1^). Furthermore, we take *β*=*μ* *R*_0_/2, and other parameters are as specified earlier.

## Results

### Variation in the duration of immunity increases the height of epidemic peaks

To investigate the impact of variation in the duration of immunity on the epidemic dynamics, we compare scenarios with and without variation in the duration of immunity. For the moment, we consider the case without vaccination. If the duration of immunity is constant, the number of individuals who are infected over an epidemic is balanced exactly by the number of individuals added to the susceptible pool during the inter-epidemic period by waning of immunity. As a consequence, we observe regular annual epidemics in this scenario (Fig. 1).

When we take the more realistic assumption that the duration of immunity is variable, and vary the fraction of the population that loses its protection in each year, the situation is different. In this case, years with no or hardly any influenza activity are interspersed with years of large influenza epidemics (Fig. 1). Susceptible individuals who escape infection in mild seasons (i.e. seasons in which by chance only a small fraction of the population loses immunity) continue to be susceptible in the next season, resulting in an accumulated pool of susceptible individuals. Importantly, the mean peak of the prevalence is larger in the variable case, while on average the same fraction of the population becomes susceptible by losing protection against the virus each season. The disproportionally large outbreaks are the result of indirect effects in the infection dynamics, namely that each additional infection increases the exposure and the probability of infection of susceptible individuals. In other words, a positive feedback of infections exists during the epidemics that cause many infections in short time when the pool of susceptible individuals is large.

### Variation in the duration of immunity reduces the impact of vaccination

Next, we incorporate vaccination into the model. We consider two types of individuals (susceptible and immune to infection), and assume a vaccination coverage (i.e. per person probability of vaccination) of 10% and vaccine efficacy of 0.5 (Methods). Hence, a vaccinated person is fully immune with probability 0.5 and fully susceptible with probability 0.5, i.e. the probability of primary vaccine failure is 0.5. This assumption is relaxed later by adding partial protection to the model.

Even in this idealized context, we find large variation in the size of epidemics in the presence of vaccination, due to variation in the duration of immunity (Fig. 1). Although the mean of the epidemic peaks is lower post-vaccination than pre-vaccination, and large outbreaks occur less frequently, vaccination cannot prevent the irregular pattern of years with small or no outbreaks interspersed with occasional large outbreaks. In contrast, if the duration of protection is constant, vaccination has a substantial protective effect in the first years after its introduction (the honeymoon period [43]), and a regular pattern of (much smaller) yearly epidemics reappears in subsequent seasons.

**Fig. 2:**
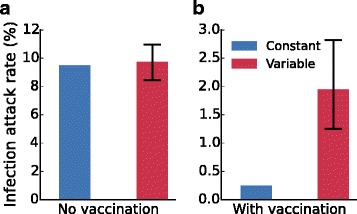
Attack rates are increased in models with variable duration of immunity. A comparison of the attack rates with a constant (blue) and a variable (red) duration of immunity without vaccination (**a**) and with vaccination (with a coverage of 20%) (**b**). With vaccination the attack rates are significantly higher in the scenario with variable duration of immunity, while in both cases the average duration of immunity is identical. Shown are median values of 1,000 simulations. Black error bars denote the 2.5−97.5 percentiles. For each scenario, the mean infection attack rate is calculated over a period of 100 years after a burn-in period of 50 years

Comparing the average attack rates in the scenarios with constant and variable duration of immunity, we find a significant difference in the post-vaccination era (Fig. 2 b). No such difference is observed without vaccination (Fig. 2 a). This is surprising, as on average immunity is lost at identical rates in both scenarios. Here, individuals who would have escaped from infection in the scenario with constant duration of immunity can be infected when the duration of immunity is short in the scenario with variable duration of immunity.

### Elevated attack rates near the critical vaccination coverage

**Fig. 3:**
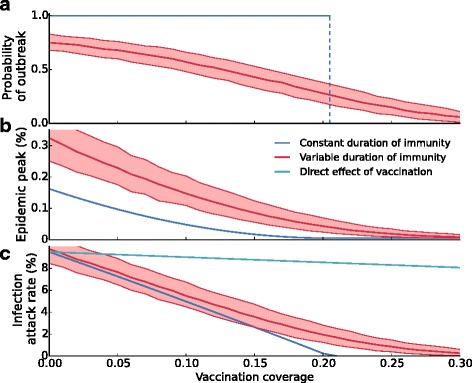
Infection attack rates and epidemic peaks are increased in models with variable duration of immunity. Shown are (**a**) the yearly probability that an epidemic will occur, (**b**) the peak prevalence in an epidemic, and (**c**) the infection attack rate, all as a function of the vaccination coverage. The figures show these quantities with constant (blue lines) and variable duration of immunity (red lines). Notice that if the duration of immunity is variable, the infection attack rate and the epidemic peak are always higher than if the duration of immunity is constant. Also notice that the difference between the infection attack rates in both scenarios is most pronounced at a vaccination coverage around the outbreak threshold (approximately at a vaccination coverage of 21%). The red line and the shaded area indicate the median and the 2.5-97.5 percentiles of 1,000 simulations, respectively. Each simulation lasts 150 years. Mean values are calculated over the last 100 years

For influenza, the vaccination coverage varies substantially over time and between countries/regions. Therefore, we investigate how the probability of an epidemic, the infection attack rate, and peak prevalence are molded by vaccine uptake (Fig. 3). As expected, in the scenario with constant duration of immunity there is a sharp transition at a critical vaccination coverage: yearly epidemics occur when the vaccination coverage is below the outbreak threshold, and no epidemics occur when the vaccination coverage is above the threshold. In the latter case, the increase in the number of immune individuals by vaccination is such that it is enough to prevent a critical accretion of susceptible individuals by waning of immunity and demographic turnover (Fig. 3 a). This does not hold anymore in the scenario with variable immunity. In this scenario, epidemics may occur even when the vaccination coverage is high, and the influx of susceptible individuals by waning immunity and demographic turnover is on average more than counterbalanced by vaccination. At low vaccination coverage no outbreaks occur in some of the years, but the average infection attack rates are comparable with those obtained in the scenario with constant immunity. In a similar vein as before (Fig. 1), we find that the peak prevalence is increased in the case of variable duration of immunity at any vaccination coverage (Fig. 3 b). Here the (absolute) difference is most pronounced if the vaccination coverage is low.

**Fig. 4:**
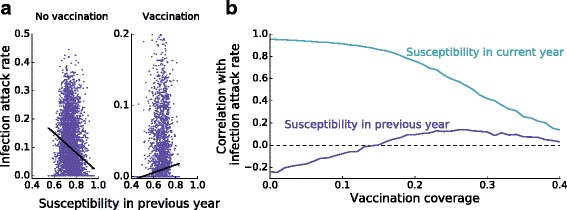
Correlations between the infection attack rate and susceptibility in the current and previous year. (**a**) The Pearson correlation between the number of susceptible individuals at the start of one influenza season and the infection attack rate in the next influenza season is negative without vaccination ($\hat {\rho }=-0.24$; p < 0.00001), as a high susceptibility in one year implies a high probability of an epidemic in that year, and a low probability in the next year. At higher vaccination coverage, however, the accumulation of susceptible persons over multiple years becomes more important, and the correlation between susceptibility in one year and the infection attack rate in the next year is reversed ($\hat {\rho }=0.13$; p < 0.00001). The switch in correlation from negative to positive is seen in (**b**) as a function of vaccination coverage (blue line). The correlation between the number of susceptible individuals at the start of the influenza season and the infection attack rate in the same year is positive, with correlations decreasing with increasing vaccination coverage (cyan line). Parameters are as in Fig. 3; in the right panel of (a) a vaccination coverage of 25% is used. For each vaccination coverage, we analyze time series of 10,000 years

A further comparison of scenarios with variable and constant immunity shows that the attack rate is higher in the scenario with variable immunity, especially when the vaccination coverage is close to the outbreak threshold (Fig. 3 c). Here the accumulated number of susceptible individuals determines to a large extent whether an outbreak occurs, and the attack rates do not depend solely on the loss of immunity since the last epidemic. This is illustrated in Fig. 4, which shows that the attack rate correlates not only positively with the susceptible fraction at the start of the current season, but also with the susceptible fraction at the start of the previous season (Fig. 4 a). In this regime, i.e. above a vaccination coverage of 15% (Fig. 4 b), epidemics occur if enough susceptible individuals have accumulated over multiple seasons. Notice that in the absence of vaccination, the correlation between the infection attack rate and susceptibility in the previous season is negative (Fig. 4 a), because in this case a high susceptibility at the start of the influenza season is likely to result in an outbreak in the same season, making the occurrence of an outbreak in the next season less likely.

Thus, the analyses show that a variable duration of immunity changes the epidemiological dynamics in two ways. First, epidemics cease to occur every year, while the size of epidemic peaks becomes highly variable and on average higher than in the constant immunity scenario. Second, the accumulation of susceptible individuals over multiple seasons in the variable immunity scenario is able to lift susceptibility over the outbreak threshold where this would not be possible in the corresponding scenario with fixed duration of immunity. Notice that these phenomena only occur in a multi-season framework, and are a consequence of the fact that the final size function is convex near the outbreak threshold (Additional file 1: Figure S1). We argue that these findings hold in general, and do not depend on specific modeling assumptions (Additional file 1: Text S1).

The phenomenon of increased attack rates in models with variable duration of immunity disappears in models in which vaccination induces protection for at most one season. Such scenarios may not be wholly unrealistic [44]. In this case, the mean epidemic infection attack rates are identical in the models with fixed and variable duration of immunity over the full range of vaccination coverages (Additional file 1: Figure S3c), the reason being that such a large fraction of the population is vaccinated around the critical vaccination coverage that no matter how much immunity is lost, an outbreak remains unlikely.

### Tailored epidemic models

More detailed models are commonly used to study influenza transmission dynamics [18, 20, 23, 33, 35]. We therefore investigate an epidemic model with additional layers of complexity to study the robustness of the above results (Methods). First, we assume Erlang (Gamma) distributed latent and infectious periods [35]. Second, we stratify the population into three age classes: children (less than 10 years old), adults (10–60 years), and elderly (older than 60 years). This extension captures the essence of heterogeneity in contact patterns by age [40–42], in particular the observation that contacts are intense among children, intermediate among adults and between adults and children, and low with and among the elderly. To be able to compare results with the earlier models without age-structure, we assume indiscriminate vaccination, i.e. vaccination coverage is equal in all age groups. Third, since influenza vaccines provide partial protection to infection [12–14], we extend the model to account for the leakiness of the vaccine. With this assumption, heavily exposed vaccinated individuals are more likely to be infected than vaccinated individuals who are sporadically exposed.

**Fig. 5:**
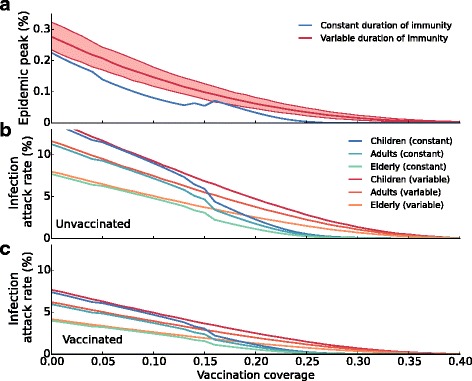
Increased attack rates in an age-specific epidemic model. Variation in duration of immunity increases the epidemic peak (**a**) and the attack rates in the unvaccinated (**b**) as well as in the vaccinated (**c**) subpopulations. In this model we assume a leaky vaccine, use age-specific contact patterns, and take realistic latent and infectious periods (using two latent compartments and two infectious compartments). The time series shows periodic behavior with periods of 2 and 3 years (at vaccination coverages 0-13% and 14-15%, respectively) for the scenario with constant duration of immunity. Shown are the averages of the periodic epidemics, explaining the irregularity of the blue curves. Percentile bands are not drawn in b and c

In the tailored model, we find that the epidemic peak as well as the attack rates are still increased when the duration of immunity is variable (Fig. 5). This is true for all age groups, and for the unvaccinated as well as vaccinated subpopulations. In line with the heterogeneity in contact patterns, and in agreement with incidence data [1], we find that the attack rates are highest in children, followed by adults and the elderly. When the vaccination coverage is such that the population is close to the outbreak threshold in the constant immunity scenario (20–30%), attack rates are higher with than without a variable duration of immunity, like we observed in the basic model (Fig. 3). Specifically, at a vaccination coverage of 25% the attack rate in unvaccinated children is only 0.5% if the duration of immunity is constant, while it is 2.8% (95% CI: 1.8%, 4.1%) if the duration of immunity is variable (Fig. 5). Similarly, at 25% vaccination coverage only 0.3% of the vaccinated children are infected in the model with a constant duration of immunity, while 1.5% (95% CI: 1.0%, 2.2%) of the vaccinated children are infected if the duration of immunity is variable. Similar results are obtained when the basic model is extended with either a leaky vaccine, or an age-dependent transmission framework (Additional file 1: Figure S4). Together, the extensions of the basic model illustrate that the finding of increased attack rates due to a variable duration of immunity is generic, and is expected to be found in even more complex models.

## Discussion

The effectiveness of humoral immune responses developed during past influenza infections to current strains is determined by the antigenic evolution of the virus [6–8]. Typically, the effective duration of immunity is relatively short but longer than a single influenza season, so that past influenza epidemics influence the epidemiology of the present epidemic. It is also known that the period of immunity varies substantially, and is driven by antigenic evolution of the virus. Here we have shown that linking the year-to-year epidemiology and including variation in the duration of immunity in transmission models together are expected to have a profound impact on the transmission dynamics of influenza. Specifically, the total size of epidemics becomes highly irregular, with increased mean peak prevalence and reduced impact of vaccination on infection attack rates.

These phenomena are not only observed in overly simple influenza models, but also in more realistic ones that take important aspects of influenza epidemiology into account. For instance, our findings are preserved in models that distinguish between infection- and vaccination-induced immunity, include both all-or-nothing and leaky immunity, and take age-structure and the essence of human contact patterns into account. In fact, the results stem from the non-linear relation between population susceptibility and infection attack rate (Additional file 1: Text S1), a hallmark of the transmission dynamics of infectious diseases. Hence, it is expected that the above findings will persist in still more complex models. Such extensions could for instance include an explicit distinction between influenza A and B subtypes, heterogeneity in contact rates, or explicit strain dynamics resulting in buildup and loss of immunity [45].

Several limitations and simplifications deserve scrutiny. First, in our analyses we assumed that vaccine efficacy (i.e. primary vaccine failure in our model) is a fixed quantity that is uncorrelated with other model parameters. This is unlikely to be true in practice. For instance, it seems plausible that vaccine efficacy is correlated with the rate of waning immunity, because a highly drifted virus will result in a poor vaccine match. The implication is that the tendency for overshooting in models with variable immunity may be increased further. As a consequence, our results should be conservative, in the sense that they provide a lower bound for the differences in attack rates in models with fixed and variable duration of immunity.

Another potential limitation is that we did not include blunting of antibody immune responses, which has been reported when using one vaccine strain for multiple seasons ([46] and references therein). This is done partly because this finding is still somewhat contentious, while the potential quantitative impact remains uncertain. Nevertheless, it may well be that variation of influenza epidemics between years may be due in part to this phenomenon. Hence, immune blunting could contribute to decreasing vaccine effectiveness, not only directly but also indirectly by increasing variation in infection attack rates. In fact, we argue that any mechanism that increases variation in infection attack rates is expected to reduce the impact of vaccination.

A final simplification worth mentioning is that we assumed that vaccination, the influenza epidemic, and demographic turnover and virus evolution take place on different timescales. Although this seems a reasonable assumption, timescales are not entirely separated. In particular, it is known that influenza evolves during the influenza season, and that the effectiveness of vaccination may wane during the influenza season [44, 47]. Unfortunately, it is not possible to assess the potential quantitative impact of this in the current model, which is built around the assumption that timescales can be separated.

Despite the importance to use a multi-year framework with a variable duration of immunity to describe the epidemiology of influenza and estimate the impact of vaccination, epidemiological studies invariably treat influenza epidemics as if they are independent of one another (reviewed in [15]). This has potential implications for evaluations of vaccine effectiveness, if only because the effectiveness is expected to be lower if by chance the epidemic in a certain year is larger than average, thereby increasing differences in the probability of infection in the unvaccinated and vaccinated subpopulations. Again, this is caused by the non-linearity in the infection dynamics. For instance, vaccinated persons who escape infection in a mild influenza season affecting a small fraction of the population may well be infected in a severe influenza epidemic affecting a larger fraction of the population, even though their level of immunity and match of the vaccine with the virus is identical in both cases. Thus, the epidemiological history in the population needs to be taken into account when estimating the vaccine effectiveness, for example by including the size of past epidemics as covariate in statistical analyses.

With regard to public health implications it is noteworthy that some countries have started vaccination of healthy children to reduce influenza circulation [2, 3], relying on predictions by models with a fixed duration of immunity [18, 20, 23, 33, 35]. Especially in children, however, the long-term effects of vaccination are unknown. Moreover, by increasing the vaccination coverage, it is conceivable that a situation will be created in which the pool of susceptible persons increases gradually over the years until a large epidemic occurs that affects an unexpectedly large fraction of the population.

## Conclusions

Our models show that variation in the duration of immunity negatively affects the effectiveness of vaccination of epidemic pathogens. Such variation is well-documented for influenza A, and is caused by the virus’ irregular evolution. Hence, we call for a multi-year perspective in analyses of the transmission dynamics and vaccine effectiveness of influenza.

## Additional file

Additional file
**Text S1** Provides support for the finding that increased attack rates are independent of model details. **Figure S1** Attack rates are higher when susceptibility varies. **Figure S2** Attack rates increase with increasing variation in susceptibility. **Figure S3** No increase in infection attack rates with one year vaccine-induced immunity. **Figure S4** The impact of variable duration of immunity in two extended infection disease models. **Figure S5** Variable duration of protection results in increased attack rates in the leaky vaccine model. **Figure S6** Impact of variable duration of immunity on age-specific infection dynamics. (PDF 185 kb)
