# Supplementary material for: Variation in loss of immunity shapes influenza epidemics and the impact of vaccination
Source: BMC Infect Dis. 2017 Sep 19;17:632. doi: 10.1186/s12879-017-2716-y (PMC5606000; doi:10.1186/s12879-017-2716-y)
Supplement: Additional file — Text S1 Provides support for the finding that increased attack rates are independent of model details. Figure S1 Attack rates are higher when susceptibility varies. Figure S2 Attack rates increase with increasing variation in susceptibility. Figure S3 No increase in infection attack rates with one year vaccine-induced immunity. Figure S4 The impact of variable duration of immunity in two extended infection disease models. Figure S5 Variable duration of protection results in increased attack rates in the leaky vaccine model. Figure S6 Impact of variable duration of immunity on age-specific infection dynamics. (PDF 185 kb) [file 12879_2017_2716_MOESM1_ESM.pdf]

Supplementary Information:
Variation in loss of immunity shapes influenza epidemics and the impact of vaccination

Rutger G. Woolthuis, Jacco Wallinga, and Michiel van Boven

Increased Attack Rates Are Independent of Model Details

Here we argue that the increase of infection attack rates observed in the models considered above are generic, and can be explained by the shape of the attack rate function that relates the susceptibility before an epidemic to the infection attack rates over the course of the epidemic.

Let $Z(s)$ be the attack rate as function of the susceptibility s (Eq. 2). $Z(s)$ is zero for low susceptibility below the critical susceptibility $s^* = 1/R_0$ and positive and increasing elswhere (see blue curve in Figure S1). The function $Z(s)$ is concave if $s > s^*$. This shape is characteristic for any attack rate function and is similar for different values of the reproduction number. When the susceptibility varies from year-to-year, we can distinguish three cases which describe the possible effects of a variable susceptibility:

- *Case I,* $s < s^*$ *for all s:*

  The susceptibility is always subcritical, and no major outbreaks can occur. Here $E[Z(s)] = Z(E[s]) = 0$, with $E[.]$ denoting expectation.

- *Case II,* $s > s^*$ *for all s:*

  The susceptibility is always supercritical. Since the attack rate function is concave if $s > s^*$, we have $E[Z(s)] \leq Z(E[s])$ by Jensen's inequality, and variation in susceptibility is expected to lower the infection attack rate.

- *Case III,* $s \approx s^*$*:*

  In some years the susceptibility is subcritical, and in other years it is supercritical. When $E[s] < s^*$, it is clear that a variable susceptibility results in a larger or equal attack rate compared to a constant susceptibility, i.e. $E[Z(s)] \geq Z(E[s]) = 0$.

  In the case $E[s] > s^*$, the attack rate around the discontinuity at s^* can be approximated by a continuous, convex function (going from slope 0 below s^* to slope 2 above s^*). By Jensen's inequality this yields $E[Z(s)] \geq Z(E[s])$.

For the dynamics of influenza over many years, case III is most relevant for the following reason. The disease dynamics is such that s tends to hover around s^*, since on the one hand

epidemics decrease s from above the threshold to below the threshold, while waning of immunity and demographic turnover increase s until it is above the threshold again. Thus, for an endemic virus like influenza, s will be around s^* most of the time.

Case II is outside the regime of interest, as for low R_0 the attack rate equation is only modestly concave on the interval $s^* < s < 1$. This is shown for R_0 = 1.4 and R_0 = 2.8 in Figure S1, where at high s no difference is visible between the scenarios with a constant duration of immunity and a variable duration of immunity.

Notice that the higher the variability in the susceptibility, the more the attack rates are increased. This is seen in Figure S1 as well as in Figure S2. In the latter figure, we assume $s \sim N(\mu_s, \sigma)$, and investigate the effect of increasing σ keeping the mean fixed at the critical susceptibility ($\mu_s = s^* = 1/R_0$). Notice also that the level of increase in the attack rates does not depend on R_0 as long as σ is not too large. Thus, the level of variability in the duration of immunity determines the increase in the attack rate at $s \approx s^*$.

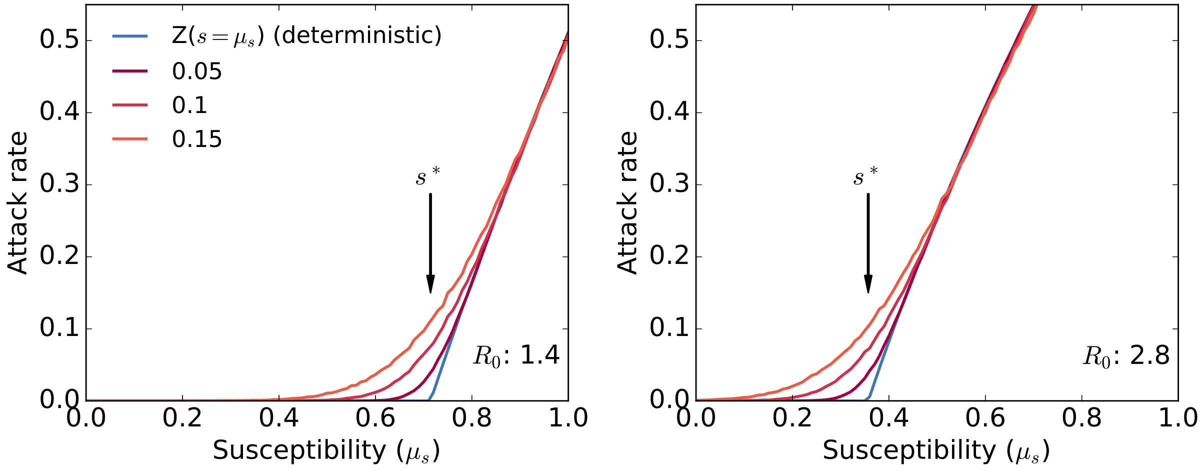

Figure S1: Attack rates are higher when susceptibility varies. Comparison of attack rates as a function of a fixed susceptibility $s = \mu_s$ or a normally distributed susceptibility ($s \sim N(\mu_s, \sigma)$, for σ = 0.05, 0.10, and 0.15) for $R_0 = 1.4$ (a), and $R_0 = 2.8$ (b). Around the critical mean susceptibility $\mu_s = s^* = 1/R_0$ the attack rates are higher in the case that the susceptibility is stochastic. Notice that for these R_0 values the attack rate function is slightly concave at high μ_s (case II, see Supplementary text), such that the attack rates in the deterministic and stochastic cases are similar for μ_s far greater than s^*.

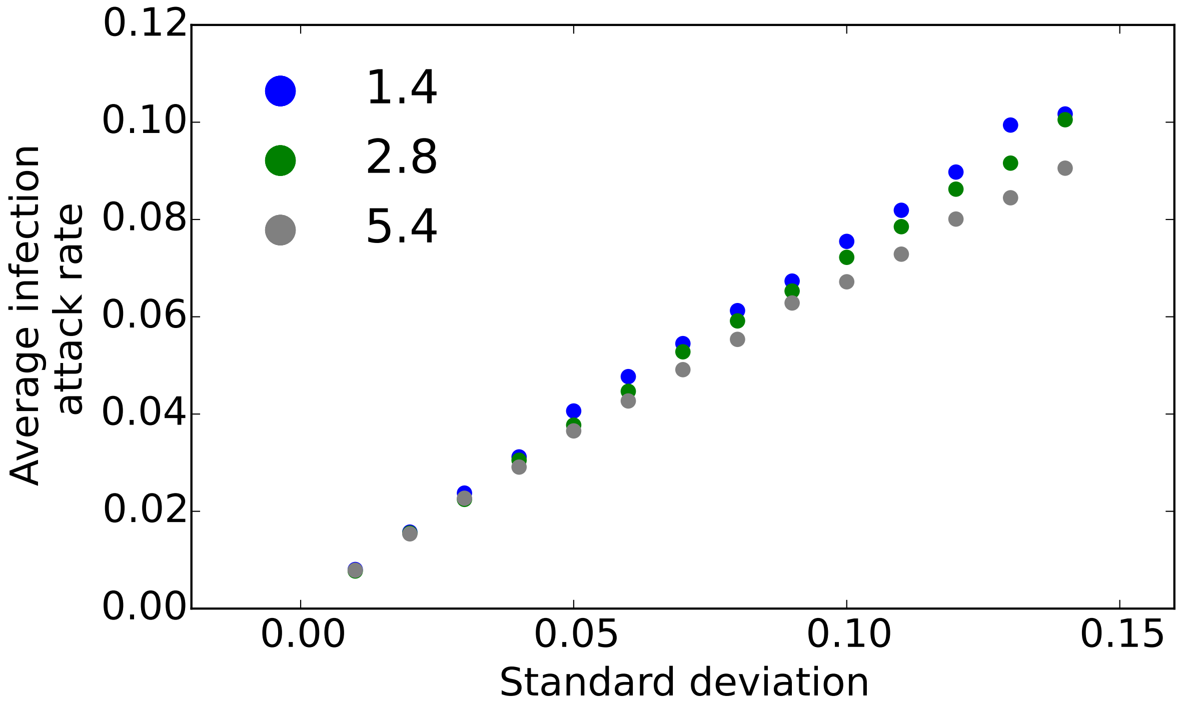

Figure S2: Attack rates increase with increasing variation in susceptibility. Shown are the attack rates as function of the standard deviation (σ) in the susceptibility using a normal distribution around the critical susceptibility ($N(\mu_s = s^*, \sigma)$) for various reproductive numbers (R_0).

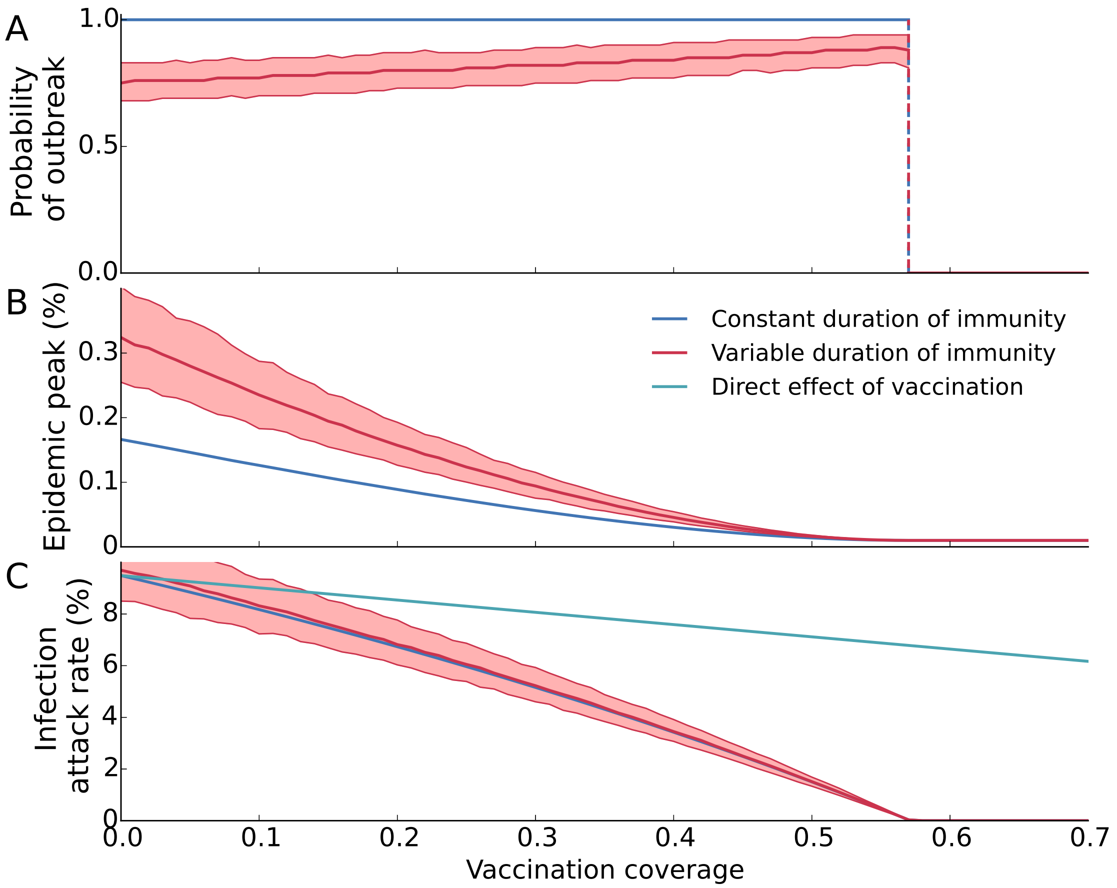

Figure S3: No increase in infection attack rates with one year vaccine-induced immunity. Shown are (a) the yearly probability that an epidemic will occur, (b) the peak prevalence, and (c) the infection attack rate, all as a function of the vaccination coverage. We present these quantities with constant (blue lines) and variable duration of immunity (red lines). In comparison with Fig. 3, the probability of an epidemic is higher, and there is a sharp vaccination threshold that determines whether or not epidemics are possible. As in Fig. 3, the epidemic peaks are increased, especially at low vaccination coverage. The average infection attack rates, however, are now equal in the scenarios with fixed and variable duration of naturally acquired immunity. Notice that the critical vaccination coverage is much higher than in Fig. 3 due to the short period of protection.

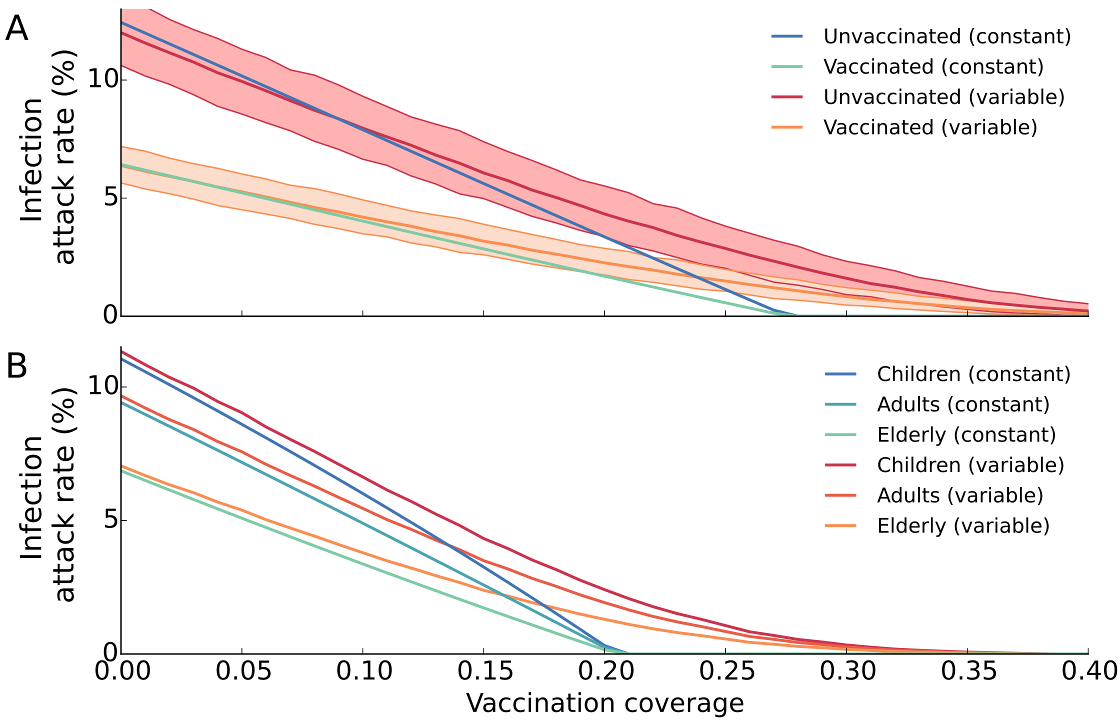

Figure S4: The impact of variable duration of immunity in two extended infection disease models. Shown are the infection attack rates with a leaky vaccine (a) and with age-specific contact patterns (b) for the scenarios with a constant (blue) or variable (red) duration of immunity. As in the basic model (Fig. 3), more realistic models with a leaky vaccine (a) and age-dependent heterogeneity in transmissibility (b) show increased attack rates around the critical vaccination coverage due to a variable duration of immunity. This is true for the (un)vaccinated populations (a) and for all age groups (b). The age-specific model is stratified in children (<10 years), adults (10-60 years), and elderly (>60 years). Attack rates are calculated as percentages of the subpopulations. For clarity we omit the 2.5-97.5 percentiles for the variable duration of immunity in (b), but see Figure S6.

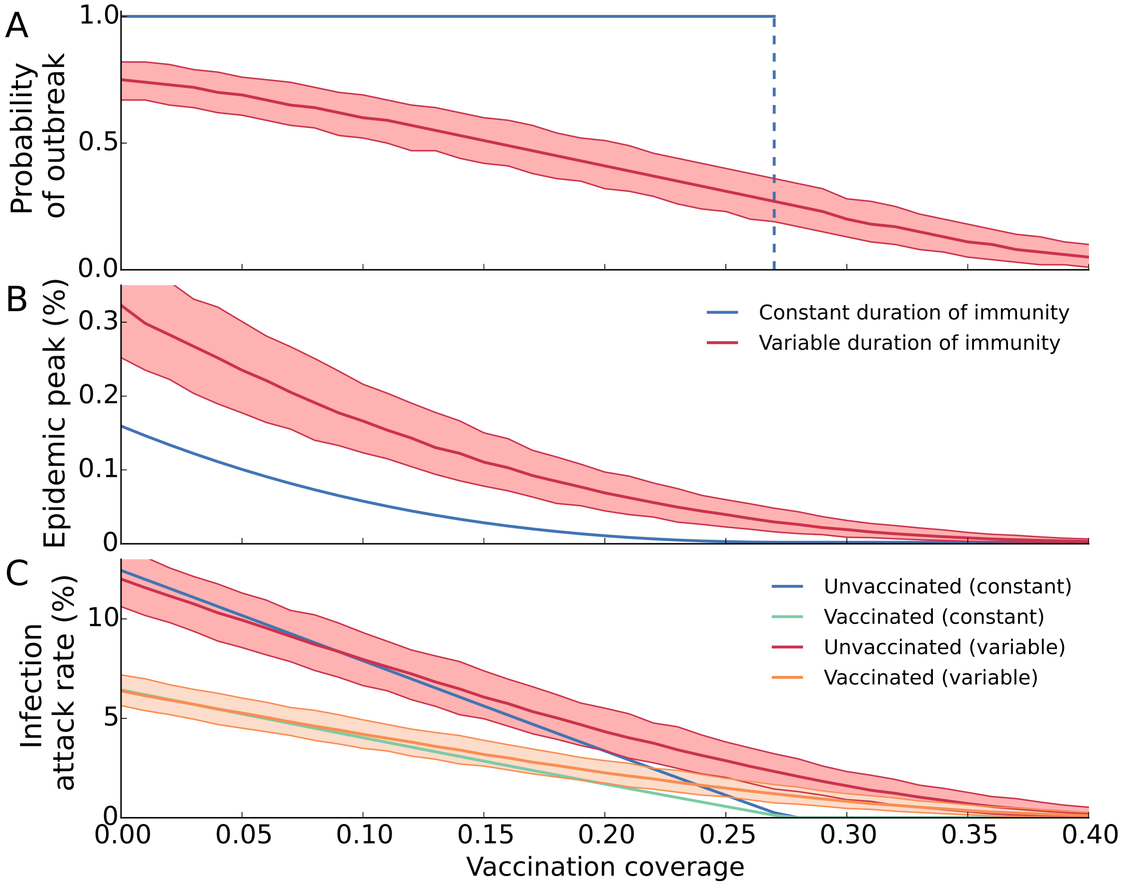

Figure S5: Variable duration of protection results in increased attack rates in the leaky vaccine model. For the unvaccinated and vaccinated population, we depict for a range of vaccination coverages (a) the yearly probability that an epidemic will occur, (b) the peak prevalence in an epidemic, and (c) the infection attack rate. We distinguish scenarios with constant (blue/cyan lines) and variable duration of immunity (red/orange lines). Especially around the critical vaccination coverage, attack rates are higher due to a variable duration of immunity in both, the unvaccinated and the vaccinated, populations (c) similarly as in Fig. 3. The epidemic peaks are also more pronounced in the leaky vaccine model if the duration of immunity is variable. Attack rates are calculated as percentage of the (un)vaccinated susceptible population (contrasting the attack rates in Fig. 3which are calculated as percentage of the total population). Notice that (c) is also shown in b.

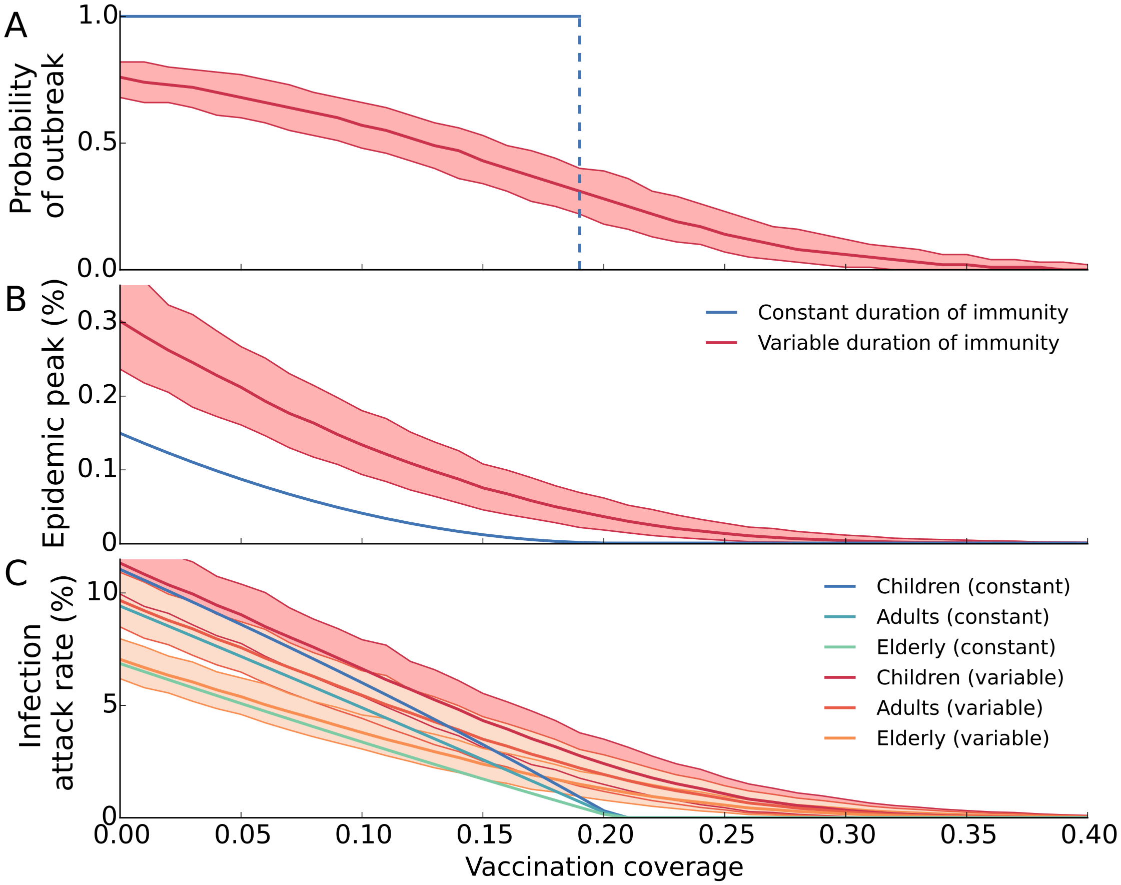

Figure S6: Impact of variable duration of immunity on age-specific infection dynamics. The probability of an outbreak (a), peak prevalence (b), and attack rates (c) for children (<10 years), adults (10-60 years), and elderly (>60 years) show similar behavior as in the main model (Fig. 3), i.e. the existence of a large range of vaccination coverages with non-zero probability of outbreak and increased peaks of the prevalence in the case of a variable duration of immunity (red) compared to a constant duration (blue). The attack rates are increased in all age groups due to the variable duration of immunity (red-orange), compared with the scenario wherein the duration of immunity is constant (blue-green). Due to a high contact rate in children and a low contact rate for elderly, the attack rate is the highest in the youngest age group and the lowest in the group of elderly. Attack rates are given as percentages of the indicated age groups. Notice that (c) is also shown in Figure S4c without credibility intervals.
